# Annotations

**Published:** 1893-12-02

**Authors:** 


### Annotations.

### Public School Discipline.

About the middle of last month the following statement was published in the press:—

Parents and Schoolmasters.—Some months ago an assistant master at a large public school administered summary chastisement to a scholar by a series of severe boxes on both ears, with the result that the drum of one of the ears was burst. The parent, after bringing the matter before the governing body, and not considering their reply satisfactory, brought an action against the master in the Court of Queen's Bench for assault. The master expressed his deep regret and paid a sum into court, which has been accepted. The case has been more than once referred to in the press, and has excited considerable interest. The boy remains at the school.

The details of the case in question are known to members of the medical profession and to many parents. The master referred to is an able and devoted servant of one of the great public schools, whose labours have materially aided its development and success. He is popular with the boys, has given the best years of his life to the school, and his services are in many ways indispensable to its well-being and progress. Unfortunately he has a very hasty temper, which has more than once led him to commit acts which have ended disastrously to more than one of the boys as well as to himself. There can be no doubt that every headmaster ought to insist that no other master should administer corporal punishment to any boy at the time the offence is committed, or at all, unless he is deputed to do so by himself. The best discipline is secured by vesting corporal punishment in the hands of the headmaster alone. Any under master who strikes a boy in anger should be suspended, and if the offence is repeated there can be no question that he ought to be removed. In the case under consideration the assault was committed, we understand, out of school hours, and the boy who was injured had in reality committed no offence of any kind. We think the parents have shown great forbearance in the present case, and that the governors would have been better advised in the interests of the master and the school if they had relieved him of all teaching responsibility, while encouraging him to retain his other offices for which he is pre-eminently qualified.

### A Poor Man's Church.

We have received the report of the church of All Hallows, East India Docks. The parish of All Hallows numbers 10,000 souls. Some of these doubtless belong to the Roman Catholic and Nonconforming Churches; but in all communities, especially in all poor communities, there are always a large number who belong to the Established Church—or to none. It is the province of the Established Church, and it is one of the arguments for keeping an Established Church, that it is its province to look after those who belong to no other. That this is done in All Hallows the weekly time-table, which shows how every hour of every day, from seven in the morning until ten at night, is occupied in work, abundantly proves. But the parish is a poor one, and its means are not equal to its needs. The income of the living is £420, and there is no vicarage. But one man cannot do all the work of so crowded a parish; there are three curates, to whose support Mr. Dalton, the vicar, contributes £120 a-year. The rest has to be met by voluntary contributions, mostly from friends outside the parish. There are, within the boundaries of All Hallows, some large employers of labour, but these, as Mr. Dalton says, are all "companies," and do not seem to feel that in their corporate capacity they are bound to provide for the spiritual welfare of those they employ. Only one subscription comes from this source—£4 per annum from a shipping company. This will not do much towards providing for all the agencies of the church. By these we do not mean the services. The cost of these, though there is service twice daily, and the church is always open for private prayer, is met by the offertories. But there are two mission-rooms, which are always in use for Bible classes, children's services, &c. There are also maintained clubs for men and boys, a soup kitchen, a penny bank, Bands of Hope, mothers' meetings, Sunday-schools, and many other forms of philanthropic work. The rescue and preventive work is of great importance, but unfortunately the fund for it now shows a deficit; so does the relief fund for sickness, so does the curate fund, so does the mission-room, so does the parish-room. None of the debts are large; the total amount is £128 17s. 8d. But the money is owing, not to tradesmen, but to the vicar, who has kept the work going out of his own pocket. But he cannot do this for ever, and he appeals for money to keep up the agencies which have been established. The total cost is £1,400 a-year—no such great sum to be drawn from wealthy London! If only the shareholders in some of the local companies would spend a fragment of their dividends on the spiritual welfare of those they employ! We would conclude by quoting some of Mr. Dalton's own simple words: "I cannot help feeling with greater force every year how serious this matter of finance is. Here I am responsible for a parish of 10,000 souls, and instead of being free to devote all my time to the spiritual and other work of the parish, I have always to be begging, begging, begging. . . . I am *anxious*, for unless this appeal calls forth a liberal response, some department of work must be cut down, or I must hand over the parish to some one possessed of more worldly goods," We ask our readers to prevent this severance by sending such contributions as they can afford to the Rev. A. E. Dalton, All Hallows Clergy House, 411, East India Dock Road, Poplar, E.

### Schoolboy Students.

"My son isn't fifteen yet, and he has passed the preliminary medical examination; he will begin his classes next session." This remark was recently made by a fond mother, and the good lady had not the slightest idea of the horror which she raised in the breast of at least one of her hearers. Doubtless the boy was clever, and, having shot through school life with telegraphic rapidity, had acquired the modest quantity of classical and mathematical knowledge requisite for the medical preliminary; but this would hardly imply that he was fit to commence the study of medicine. In the first place, the method of tuition is so different that a boy who might do well under the careful tutoring, not to call it cramming, of a schoolmaster, might not be skilled in distinguishing essentials from details in instruction given by means of lectures. It requires a mind which has attained some critical faculty to select and arrange the facts a lecturer sets before it—unless, indeed, the lecturer be like that amiable and accurate German professor who would pause in his discourse to say, "Here should the gentlemen a comma put." Similarly with books; the school-books of our day are graded, annotated, explained, until an intelligent passivity becomes the most profitable attitude for the pupil; he has had no training for the discrimination and careful observation which medical studies demand; his mind is scarcely mature enough to possess these qualities; yet the books he has to study from are not such as can be "got up" by memory without the aid of judgment. Moreover, medicine is a science of humanity; it must be studied in a spirit of reverence which is not too often found in lads, and we may arouse prurient instead of scientific thoughts in the minds of young men by too soon opening up to them the mysteries of life and death. Finally, as an argument for those who may think this notion fantastic and immaterial, the time gained at the beginning of the youth's career is often lost at the end. Patients will not trust too young a doctor. A course of logic and physical science would fill the time profitably between the end of school life and the beginning of medical study.

